# Socioeconomic inequalities in life expectancy within and between native-born and foreign-born populations: a comparative study of 10 European countries

**DOI:** 10.1093/ije/dyag045

**Published:** 2026-04-17

**Authors:** Su Yeon Jang, Frank J van Lenthe, Mikko Myrskylä, Anna Oksuzyan, Silvia Loi, Matthias Bopp, Henrik Brønnum-Hansen, Patrick Deboosere, Ramunė Kalėdienė, Mall Leinsalu, Pekka Martikainen, Olof M Östergren, Sophie Psihoda, Enrique Regidor, Nicolás Zengarini, Wilma J Nusselder

**Affiliations:** Max Planck Institute for Demographic Research, Rostock, Germany; Department of Public Health, Erasmus MC University Medical Center Rotterdam, Rotterdam, Netherlands; Oxford Institute of Population Ageing, University of Oxford, Oxford, United Kingdom; Department of Public Health, Erasmus MC University Medical Center Rotterdam, Rotterdam, Netherlands; Max Planck Institute for Demographic Research, Rostock, Germany; Helsinki Institute for Demography and Population Health, University of Helsinki, Helsinki, Finland; Max Planck—University of Helsinki Center for Social Inequalities in Population Health, Rostock, Germany; Max Planck—University of Helsinki Center for Social Inequalities in Population Health, Helsinki, Finland; Max Planck Institute for Demographic Research, Rostock, Germany; School of Public Health, Bielefeld University, Bielefeld, Germany; Max Planck Institute for Demographic Research, Rostock, Germany; Max Planck—University of Helsinki Center for Social Inequalities in Population Health, Rostock, Germany; School of Geography, University College Dublin, Dublin, Ireland; Epidemiology, Biostatistics and Prevention Institute, University of Zurich, Zurich, Switzerland; Department of Public Health, Faculty of Health Sciences, University of Copenhagen, Copenhagen, Denmark; Department of Sociology, Brussels Institute for Social and Population Studies, Vrije Universiteit Brussel, Brussel, Belgium; Department of Health Management, Lithuanian University of Health Sciences, Kaunas, Lithuania; Stockholm Centre for Health and Social Change, Södertörn University, Huddinge, Sweden; Department of Epidemiology and Biostatistics, National Institute for Health Development, Tallinn, Estonia; Max Planck Institute for Demographic Research, Rostock, Germany; Helsinki Institute for Demography and Population Health, University of Helsinki, Helsinki, Finland; Max Planck—University of Helsinki Center for Social Inequalities in Population Health, Rostock, Germany; Max Planck—University of Helsinki Center for Social Inequalities in Population Health, Helsinki, Finland; Department of Public Health Sciences, Stockholm University, Stockholm, Sweden; Directorate Social Statistics, Statistics Austria, Vienna, Austria; CIBER Epidemiología y Salud Pública (CIBERESP), Madrid, Spain; Epidemiology Unit, ASL TO3 Piedmont Region, Collegno (Torino), Italy; Department of Public Health, Erasmus MC University Medical Center Rotterdam, Rotterdam, Netherlands

**Keywords:** life expectancy, country of birth, education, migrants, Europe

## Abstract

**Background:**

Foreign-born residents in high-income countries often outlive the native-born population, but it remains unclear how this advantage varies across countries and socioeconomic groups. We aimed to assess socioeconomic inequalities in the life expectancy advantage of foreign-born populations across 10 European countries.

**Methods:**

Using national population registers and census, we collected data on mortality by country of birth and educational attainment in 10 European countries from 2010 to 2019. Based on these data, we estimated partial life expectancy between ages 35 and 80 years for native-born and foreign-born populations in each country, both overall and by education. We then decomposed the overall gap between native- and foreign-born populations into differences in education-specific mortality and the educational composition of the population.

**Results:**

Foreign-born populations had higher partial life expectancies than native-born populations in all countries except Sweden and Estonia, especially among individuals with low-level education. Decomposition analyses revealed that the overall longevity advantage of foreign-born populations was largely attributable to their lower mortality in less-educated groups. However, in most countries—except Estonia, Lithuania, and Spain—the overrepresentation of less-educated individuals among the foreign-born partially cancelled out these advantages.

**Conclusions:**

The longevity advantages among foreign-born populations in European countries may reflect socioeconomic barriers that concentrate relatively healthy, resource-rich immigrants into lower socioeconomic groups. Improving how societies recognize and use the skills and resources of foreign-born populations can help reduce these barriers, benefiting their longevity outcomes and contributing to more inclusive societies.

Key Messages

- We examined how socioeconomic inequalities shape the life expectancy advantage of foreign-born populations across 10 European countries.
- Foreign-born populations had longer life expectancy than native-born populations across most European countries, especially among those with low education, although the overall advantage was reduced in countries where foreign-born populations were overrepresented in lower socioeconomic groups.
- Our findings provide cross-national evidence that the concentration of foreign-born populations in lower socioeconomic groups can structurally reduce their overall longevity advantage, underscoring the importance of addressing barriers to education and social advancement they face.

## Introduction

Foreign-born residents in high-income countries worldwide often outlive native-born populations despite their relatively low socioeconomic status [1–6]. The longevity of foreign-born populations appears to challenge the well-established pattern of wealthier and better-educated populations living longer [7]. Scholars widely attribute this mortality paradox to the ‘healthy immigrant effect’, which postulates that foreign-born populations generally consist of selected individuals who are sufficiently healthy to relocate to another country [8, 9].

This explanation, however, leaves open an important question: Does socioeconomic disadvantage still matter for the longevity of foreign-born populations? Prior research addressing this question has shown that the mortality gap between low- and high-socioeconomic groups is smaller for foreign-born than for native-born individuals [10–13]. This ‘flatter’ gradient may arise from relatively lower mortality among foreign-born individuals with low socioeconomic status due to stronger health selection, weaker impacts of low status carried over from the country of origin, cultural and behavioural protections, or data artefacts [12–17]. It may also reflect reduced survival benefits among foreign-born individuals with higher socioeconomic status, as they often encounter structural barriers that constrain the advantages typically associated with higher education and income [18, 19].

Notably, the importance and impact of these mechanisms likely vary across countries, as multiple contextual factors, including migration histories, welfare, and healthcare systems, shape the extent to which socioeconomic disadvantage translates into mortality [20]. A systematic description of how socioeconomic inequalities in life expectancy among foreign-born populations differ across countries is therefore needed to better characterize these patterns. Yet, this type of analysis has so far been limited, primarily due to data constraints.

Europe provides an ideal setting for such a description, offering institutional diversity that has contributed to substantial cross-national differences in socioeconomic inequalities in mortality [21–24]. At the same time, European countries differ in their migration histories, resulting in varied socioeconomic compositions of their foreign-born populations. For instance, despite a recent increase in highly educated immigrants, the United Kingdom, the Netherlands, and Sweden have historically received many low-educated manual workers from either former colonies or neighbouring countries [25], which may explain the relatively lower life expectancies observed among their foreign-born populations [26, 27].

In the current study, we examined life expectancy among native-born and foreign-born populations across 10 European countries by socioeconomic status (as measured by education), made possible by a newly collected longitudinal dataset. Our aim was to assess whether socioeconomic inequalities exist in the life expectancy advantage of foreign-born populations in Europe and, if so, how these inequalities contribute to the overall differences in life expectancy between the foreign-born and native-born populations. To this end, we employed partial life expectancy (PLE), also known as temporal life expectancy, a useful metric for uncovering systemic mortality inequalities [28]. Our comparative analyses can help to identify countries and socioeconomic contexts where foreign-born populations face disadvantages (or reduced advantages) in mortality.

## Methods

### Data

We harmonized administrative data from 10 European countries (Austria, Belgium, Denmark, Estonia, Finland, Italy, Lithuania, Spain, Sweden, and Switzerland) between 2010 and 2019 (overview in Supplementary Table S1). The datasets covered entire national populations in most countries, except Italy (Turin only), Spain (9% sample), and Switzerland (21% sample). Country inclusion for specific analyses varied depending on data availability (Supplementary Table S2).

In each country, census or register populations were longitudinally tracked to estimate population exposure to risk (person-years)—i.e. the total time individuals resided in the country until death, emigration, or the end of follow-up—with mortality calculated as all-cause deaths divided by the corresponding person-years. These values were calculated by age, gender, country of birth, and socioeconomic position, excluding records with missing information on these characteristics. In most countries, <1% of records were excluded due to missing information, although some countries—most notably Belgium and Lithuania—had larger proportions of missing education data, especially among foreign-born individuals (Supplementary Table S3).

Based on the country of birth, populations in each country were first categorized into native-born and foreign-born groups, with foreign-born individuals further classified into those born in European and non-European countries (Supplementary Table S4). Given the heterogeneous economic and cultural profiles of non-European nations, we ran additional analyses excluding individuals from some high-income Western countries (Australia, Canada, New Zealand, and the United States). However, a combination of data access restrictions, data protection rules, and difficulties in categorizing certain origin groups prevented the required disaggregation in Finland (European versus non-European), Sweden (Western versus non-Western), and Belgium (Western non-European versus European).

Socioeconomic position was primarily measured through educational attainment (data collection methods in Supplementary Table S5), categorized into three levels: low (ISCED 0–2), mid (ISCED 3–4), and high (ISCED 5+). Notably, Finland could only identify educational qualifications obtained within its own system and did not include foreign qualifications; hence, Finland was excluded from the education-specific analyses. Additionally, we examined whether our findings hold when using occupational class as an alternative socioeconomic indicator, available in Austria, Denmark, Estonia, Finland, Lithuania, and Spain. Occupations were classified as manual, nonmanual, or other (farmers and self-employed) and defined only for individuals of working ages (35–65 years).

### Partial life expectancy

We first estimated age-specific mortality rates for 5-year age groups between 35 and 80 years, corresponding to the age range for which educational information was most reliable and comparable across countries. One major challenge in mortality estimation is the small number of deaths [29]. Given the very small number of foreign-born individuals from non-European countries identified by socioeconomic class in Estonia and Lithuania, we excluded these groups from the education- and occupation-specific analysis. We cross-checked the reliability of our mortality estimates by computing the smoothed mortality rates (Supplementary Material, Section B).

Based on the age-specific mortality, we calculated the PLE between ages 35 and 80 years by gender, country of birth, and socioeconomic position. We then estimated the absolute gap in PLE between native-born and foreign-born groups and between low and high-socioeconomic groups. We produced a European mean of life expectancy from the population-weighted average of the mortality of 10 countries using population statistics from Eurostat in 2015, the median year of the study inclusion period [30]. For all estimates, we determined 95% confidence intervals using bootstrapping based on the Poisson distribution of mortality with 1000 repetitions.

### Decomposition analysis

To determine how socioeconomic inequalities contribute to the foreign-born differential in PLE compared to the native-born populations, we employed the traditional stepwise decomposition [31]. We followed the algorithms proposed and implemented in prior work [32, 33]. This analysis separated the contributions of socioeconomic inequalities to the PLE gap between foreign-born and native-born populations into two main components. First, we assessed the contribution of the mortality gap between native-born and foreign-born populations in each of the low-, mid-, and high-education groups, reflecting how socioeconomic positions differently affect mortality between the two populations. Second, we evaluated the contributions of differences in educational compositions between the native-born and foreign-born populations to their gap in PLE, representing structural disparities in socioeconomic attainment.

## Results

### Foreign-born advantage in partial life expectancy

Table 1 presents the PLE for native-born and foreign-born populations. Across most included countries in all parts of Europe, PLE was higher among the foreign-born than the native-born population, regardless of gender. The only exceptions were among men in Sweden, where PLE was 0.1 years shorter (95% CI: 0.1, 0.2), and in Estonia, where the difference was similarly small (0.1 years shorter [95% CI: −0.2, 0.3]). The observed foreign-born advantage in PLE varied in size by gender and region of origin. In terms of gender, foreign-born men were expected to live 0.9 years longer (95% CI: 0.9, 0.9) than native-born men on average across Europe, while the foreign-born advantage was only 0.4 years (95% CI: 0.4, 0.4) among women. By region of origin, the foreign-born advantage in PLE was more pronounced among individuals born in non-European countries (men: 1.4 years higher [95% CI: 1.4, 1.4]; women: 0.6 years higher [95% CI: 0.6, 0.6]) than among those born in European countries (men: 0.5 years higher [95% CI: 0.5, 0.5]; women: 0.2 years higher [95% CI: 0.2, 0.2]). This large advantage among persons born outside of Europe remained even when taking the cultural background (i.e. Western versus non-Western) into account (Supplementary Table S6).

**Table 1:** Partial life expectancy gap^a^ between native-born and foreign-born populations.

	Native-born	Foreign-born		FB, European		FB, non-European	
	PLE	PLE	Gap^a^ (95% CI)	PLE	Gap^a^ (95% CI)	PLE	Gap^a^ (95% CI)
**Men**							
Europe (mean)	40.6	41.5	0.9 (0.9, 0.9)	41.1	0.5 (0.5, 0.5)	42.0	1.4 (1.4, 1.4)
Northern Europe							
Denmark	40.2	41.4	1.1 (1.0, 1.3)	41.1	0.9 (0.7, 1.0)	41.8	1.6 (1.4, 1.7)
Finland	39.7	41.0	1.3 (1.2, 1.5)	–	–	–	–
Sweden	41.2	41.1	−0.1 (−0.2, −0.1)	40.6	−0.6 (−0.7, −0.5)	41.9	0.6 (0.6, 0.7)
Western Europe							
Austria	40.1	40.5	0.4 (0.3, 0.4)	40.4	0.3 (0.2, 0.3)	41.5	1.4 (1.3, 1.6)
Belgium	39.7	40.7	0.9 (0.9, 1.0)	40.6	0.8 (0.8, 0.9)	40.8	1.1 (1.0, 1.2)
Switzerland	41.6	42.1	0.6 (0.5, 0.7)	42.1	0.5 (0.4, 0.7)	42.5	1.0 (0.7, 1.2)
Southern Europe							
Italy	41.2	41.5	0.4 (0.1, 0.7)	40.8	−0.4 (−0.9, 0.1)	42.0	0.9 (0.6, 1.2)
Spain	40.4	42.0	1.6 (1.5, 1.7)	41.7	1.2 (1.0, 1.4)	42.3	1.8 (1.7, 2.0)
Eastern Europe							
Estonia	36.6	36.6	−0.1 (−0.3, 0.2)	36.5	−0.1 (−0.3, 0.1)	42.2	5.6 (4.2, 6.8)
Lithuania	35.7	36.4	0.8 (0.5, 1.0)	36.4	0.7 (0.5, 0.9)	41.6	5.9 (4.5, 7.4)
**Women**							
Europe (mean)	42.5	42.8	0.4 (0.4, 0.4)	42.7	0.2 (0.2, 0.2)	43.1	0.6 (0.6, 0.6)
Northern Europe							
Denmark	41.8	42.6	0.8 (0.7, 0.9)	42.3	0.5 (0.4, 0.7)	43.0	1.2 (1.1, 1.4)
Finland	42.3	42.6	0.3 (0.2, 0.4)	–	–	–	–
Sweden	42.4	42.5	0.1 (0.1, 0.2)	42.3	−0.1 (−0.1, 0.0)	43.0	0.6 (0.6, 0.7)
Western Europe							
Austria	42.2	42.2	0.0 (0.0, 0.1)	42.2	0.0 (−0.1, 0.0)	42.8	0.6 (0.5, 0.7)
Belgium	41.8	42.3	0.5 (0.5, 0.5)	42.2	0.4 (0.3, 0.5)	42.4	0.6 (0.6, 0.7)
Switzerland	42.9	43.2	0.3 (0.2, 0.4)	43.1	0.3 (0.1, 0.4)	43.4	0.6 (0.4, 0.8)
Southern Europe							
Italy	42.6	42.8	0.2 (0.0, 0.4)	42.6	0.0 (−0.2, 0.3)	42.9	0.3 (0.0, 0.5)
Spain	42.7	43.3	0.6 (0.5, 0.7)	43.1	0.5 (0.3, 0.6)	43.4	0.7 (0.6, 0.8)
Eastern Europe							
Estonia	41.5	41.5	0.0 (−0.1, 0.2)	41.5	0.0 (−0.2, 0.1)	42.8	1.4 (0.1, 2.6)
Lithuania	41.0	41.2	0.2 (0.1, 0.3)	41.2	0.2 (0.0, 0.3)	42.0	1.0 (−0.9, 2.8)

FB, foreign-born; PLE, partial life expectancy between ages 35 and 80 years.

a Calculated as: foreign-born PLE—native-born PLE.

### Socioeconomic variations in the foreign-born advantage

**Figure 1:**
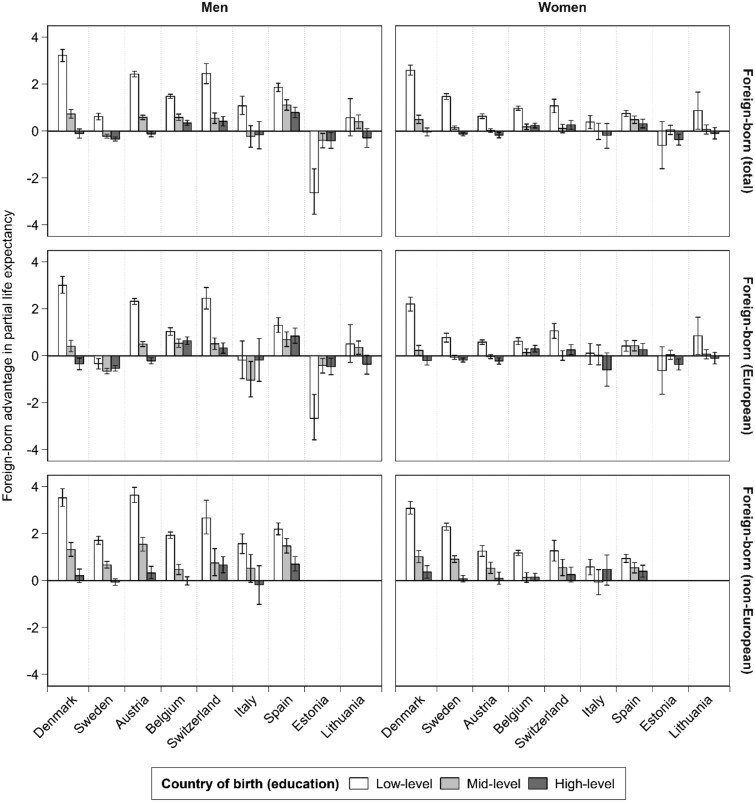
Differences in partial life expectancy between foreign-born and native-born populations in each country by education, stratified by gender and region of origin.

Figure 1 shows how the PLE differences between native-born and foreign-born populations vary across low-, mid-, and high-level education groups (detailed estimates in Supplementary Tables S7 and S8). Overall, we observed a socioeconomic gradient in the foreign-born PLE advantage, being most pronounced in the low-educated group (European mean among men: 1.6 years [95% CI: 1.6, 1.6]; among women: 0.8 years [95% CI: 0.8, 0.8]) and diminishing in the mid- and high-educated groups. This pattern, however, varied across countries. For instance, although a strong PLE advantage among low-educated foreign-born populations was observed in nearly all countries, it was absent in Estonia. Among individuals with high-level education, foreign-born populations in some countries (Belgium, Switzerland, Spain) still showed a PLE advantage, though smaller than that observed among the low-educated group, whereas the advantage was insignificant (Denmark, Italy, Lithuania) or was even reversed (Sweden, Austria, and Estonia) in others. Interestingly, individuals born in non-European countries tended to exhibit a larger PLE advantage at low education levels and a smaller disadvantage at high education levels than those born in European countries, a pattern consistently observed across all countries. The supplementary analysis using occupational class as an alternative socioeconomic indicator showed a similarly flat PLE gradient among foreign-born populations, confirming that our main conclusions are robust to the choice of socioeconomic measure (Supplementary Fig. S2).

### Decomposition of the partial life expectancy gap

Table 2 presents findings from the decomposition analysis, which disentangles how socioeconomic inequalities contribute to the overall PLE gap between native-born and foreign-born populations. Overall, we found that the foreign-born PLE advantage was largely attributable to the lower mortality among foreign-born populations with low education in all countries except Estonia. In Western European countries as well as in Sweden and Italy, however, the positive contribution of the mortality advantage among foreign-born populations—particularly among those with low-level education—on their PLE was mitigated by the educational composition of the population. Conversely, in Eastern Europe and Spain, the share of individuals with high-level education was larger in the foreign-born than in the native-born populations, contributing positively to the foreign-born differential in PLE. This general pattern held across most age groups, with the positive contribution of lower mortality among foreign-born individuals being greatest at around the sixties (Supplementary Fig. S3). Notably, however, foreign-born populations in Austria, Italy, Lithuania, and Sweden lost this mortality advantage at older ages, from age 70 onwards, likely reflecting the gradual weakening of the initial health advantage over time.

**Table 2:** Decomposition of the partial life expectancy gap between foreign-born and native-born populations.

	Contributions to the total PLE gap^a^				PLE gap^a^
	Mortality by education			Population composition	
	Low-level	Mid-level	High-level		
**Men**					
Northern Europe					
Denmark	0.84	0.29	−0.03	0.05	1.14
Sweden	0.11	−0.09	−0.10	−0.06	−0.15
Western Europe					
Austria	0.97	0.23	−0.02	−0.80	0.38
Belgium	0.73	0.14	0.07	−0.22	0.72
Switzerland	0.87	0.17	0.13	−0.59	0.59
Southern Europe					
Italy	0.48	−0.05	−0.02	−0.04	0.36
Spain	0.84	0.33	0.19	0.22	1.57
Eastern Europe					
Estonia	−0.33	−0.21	−0.09	0.56	−0.07
Lithuania	0.02	0.28	−0.06	0.52	0.76
**Women**					
Northern Europe					
Denmark	0.77	0.18	−0.01	−0.15	0.78
Sweden	0.36	0.06	−0.04	−0.25	0.13
Western Europe					
Austria	0.28	0.00	−0.03	−0.23	0.02
Belgium	0.47	0.04	0.04	−0.21	0.35
Switzerland	0.42	0.03	0.06	−0.20	0.31
Southern Europe					
Italy	0.14	0.05	−0.00	−0.02	0.16
Spain	0.33	0.15	0.07	0.05	0.61
Eastern Europe					
Estonia	−0.07	0.05	−0.09	0.11	0.00
Lithuania	0.02	0.06	−0.02	0.13	0.19

PLE, partial life expectancy between ages 35 and 80 years.

a Calculated as: foreign-born PLE—native-born PLE.

## Discussion

### Summary of findings

In this study, we found a substantial life expectancy advantage among foreign-born populations compared to their native-born counterparts across Europe, with foreign-born men living an average of 0.9 years longer (95% CI: 0.9, 0.9) and foreign-born women 0.4 years longer (95% CI: 0.4, 0.4). This advantage was particularly pronounced among those with low-level education. In nearly all countries except Estonia, Lithuania, and Spain, however, foreign-born populations had a disproportionately large share of low-educated individuals, which partially offset their overall mortality advantage.

### Interpretation of findings

Consistent with the vast literature on the migrant mortality advantage, our findings suggest that foreign-born populations generally have a longer lifespan than their native-born counterparts, particularly among men, individuals from non-European countries, and those with low-level education. One plausible explanation lies in health selection: migration inherently requires a certain level of physical, psychological, and administrative capacity that selects individuals in good health, yet these demands—and the resulting health selection—can be stronger for some groups than others [8]. For instance, immigrant men are more likely to initiate migration for work or education than women, a process that generally involves greater individual demands and therefore stronger health selection than moving for family reunification [34, 35]. Similarly, stricter entry conditions for immigrants from countries outside the European Union or Schengen area and limited socioeconomic resources among those with lower education may both require particularly good health to overcome.

However, these advantages should also be interpreted with caution, as they may be influenced by data artefacts. Although our mortality data were based on follow-ups of individuals until death or emigration, some residual over-coverage may occur if out-migrants do not deregister promptly or at all, particularly when linkages between national statistical systems are unavailable [36]. Previous evidence suggests that such over-coverage alone is unlikely to account for the foreign-born mortality advantage, yet mortality among foreign-born populations may still be underestimated to some degree [37].

One major contribution of this study lies in addressing whether socioeconomic inequalities still matter for understanding the foreign-born advantage in life expectancy. According to our findings, the generally longer lifespans of foreign-born populations can be largely attributed to the advantage among those with low education, although differences in educational composition reduce the observed advantage in most countries. This finding indicates that although low-educated foreign-born individuals experience stronger health selection [8], the concentration of foreign-born populations in this group partially offsets the survival benefits gained through selection. Our findings are particularly relevant in contexts where foreign-born populations face structural barriers to attaining higher socioeconomic positions within host societies, such as systemic discrimination and the nonrecognition of qualifications [19]. Moreover, given the opposing forces of health selection and structural barriers, future research would benefit from examining lifespan variation across educational groups among foreign-born populations.

Another important contribution of this study lies in revealing considerable heterogeneities in the observed foreign-born advantage across countries. The varying size of the foreign-born advantage across Europe presented in this study is consistent with the idea that the strength and nature of health selection underlying the life expectancy advantage among foreign-born populations depend on who migrates, from where, and under what conditions. For instance, the significant disadvantage among foreign-born men in Estonia likely reflects the legacy of Soviet-time migration, where many immigrants arrived from neighbouring countries. Having experienced intense social and economic disruption, accompanied by poverty and unhealthy behaviours such as heavy alcohol consumption, these immigrants commonly experienced elevated mortality risks [38]. As shown in our decomposition analysis, this disadvantage was largely driven by higher mortality among less-educated foreign-born individuals.

Similarly, the lower PLE among foreign-born men in Sweden compared with their native-born counterparts also reflects, at least in part, the historical context of migration to Sweden that predominantly attracted a specific group of migrants. During the second half of the twentieth century, Sweden faced a massive inflow of Finnish people as part of the Intra-Nordic labour migration, primarily composed of farmers and manual labourers [39, 40]. Consequently, the Finnish-origin population in Sweden today largely represents earlier cohorts of labour migrants with low socioeconomic status, whose persistent concentration in lower socioeconomic positions may have led to structural disadvantages that limited their potential longevity gains [41].

The decomposition analysis further revealed that the lower overall educational attainment of foreign-born populations in Western European countries (Austria, Belgium, and Switzerland) and Italy also contributed to reducing their advantage in PLE. Similar to the situation in Sweden, these disadvantaged educational profiles largely reflect the influx of many less-educated, low-skilled migrant workers, although this trend has diminished in recent years [25]. Consequently, a larger proportion of foreign-born populations faced mortality risks associated with lower socioeconomic status than native-born populations, discounting their advantage in life expectancy.

### Strengths and limitations

To the best of our knowledge, this is the first international comparative study of socioeconomic inequalities in life expectancy among native-born and foreign-born populations across Europe. This study includes a total of 10 countries across all regions of Europe in the analyses, which is by far the most extensive coverage for analyses of life expectancies among foreign-born populations. Previous comparative studies on this topic have been limited to some Nordic or Western European countries [26, 27]. Our new data adds Southern and Eastern Europe to the picture, where no previous studies have addressed the life expectancies of foreign-born populations.

Despite the opportunities associated with the inclusion of more countries, it also brings challenges related to data comparability. In particular, educational attainment was measured in different ways across the included countries. While information was generally collected through self-reported data from censuses, data from Austria, Denmark, and Sweden identified education based on official certificates. This register-based information may not be available for individuals who have obtained their degree certificates in a foreign country, leading to an underestimation of the educational attainments among foreign-born populations [16].

Another notable limitation is the lack of consideration for the duration of stay in the host country among foreign-born populations. This omission is particularly significant in countries where older and recent migrants have different mortality patterns, as exemplified by Sweden, where older migrants consisted more of Finnish people with higher mortality compared to recent arrivals from more distant countries with lower mortality. Although our age-specific decomposition partially captures these temporal differences, age serves only as an indirect and imperfect proxy for duration of stay. Without data on length of stay, we cannot disentangle these temporal effects in the foreign-born advantage in mortality and life expectancy.

In addition, part of the observed educational gradient may reflect compositional differences by migrant origin. Although descriptive checks showed that the concentration of non-European migrants in lower education groups was moderate (Supplementary Table S3), origin composition may still have contributed to the observed differences in life expectancy.

## Conclusion

Foreign-born populations in Europe generally enjoy longer lives than native-born populations, particularly in lower socioeconomic groups. However, in several countries where foreign-born populations include a high proportion of low-educated individuals, the mortality advantage tends to be mitigated by this unfavourable educational composition. Our findings may reflect socioeconomic barriers in host countries that concentrate comparatively healthy and resource-rich immigrants in lower socioeconomic groups. This concentration may create an impression that immigrants experience a smaller disadvantage in mortality than those typically associated with low socioeconomic status. Paradoxically, the overrepresentation of immigrants in lower socioeconomic groups exposes a larger proportion of foreign-born populations to mortality risks associated with lower socioeconomic status, potentially offsetting the mortality advantage. Hence, improving educational opportunities for foreign-born populations may help enhance their longevity, contributing to healthier and more inclusive societies.

## Ethics approval

This study was exempt from ethics review as it involved the analysis of secondary data and did not include any identifiable personal information.

## Supplementary Material

dyag045_Supplementary_Data

## Data Availability

Due to data protection regulations of the national register-holders providing the data, we are not allowed to make the data available to third parties.
