# Supplementary material for: Socioeconomic inequalities in life expectancy within and between native-born and foreign-born populations: a comparative study of 10 European countries
Source: Int J Epidemiol. 2026 Apr 17;55(3):dyag045. doi: 10.1093/ije/dyag045 (PMC13089551; doi:10.1093/ije/dyag045)
Supplement: dyag045_Supplementary_Data [file dyag045_supplementary_data.docx]

**Supplementary material to
“Socioeconomic inequalities in life expectancy within and between native-born and foreign-born populations:**

**A comparative study of 10 European countries”**

**Table of Contents**

[Section A: Data description 3](#_Toc221205671)

[**Table S1** Overview of harmonized data 3](#_Toc221205672)

[**Table S2** Countries included in total, education-specific, and occupation-specific estimation of partial life expectancy for native-born and foreign-born populations 4](#_Toc221205673)

[**Table S3** Distribution of person-years and deaths by country of birth status and education (%) 5](#_Toc221205674)

[**Table S4** Definition of subgroups based on the country of birth 6](#_Toc221205675)

[**Table S5** Collection of educational information among foreign-born populations in each country 7](#_Toc221205676)

[Section B: Mortality smoothing 8](#_Toc221205677)

[Methods 8](#_Toc221205678)

[Results 9](#_Toc221205679)

[**Figure S1** Differences in partial life expectancy between foreign-born and native-born populations in each country by education in samples stratified by gender and region of origin, estimated from the smoothed mortality 9](#_Toc221205680)

[References 10](#_Toc221205681)

[Section C: Supplemental findings 11](#_Toc221205682)

[**Table S6** Partial life expectancy gap between native-born and foreign-born populations from non-European countries (total non-European versus non-Western non-European) 11](#_Toc221205683)

[**Table S7** Partial life expectancy gap between native-born and foreign-born populations by socioeconomic status 12](#_Toc221205684)

[**Table S8** Socioeconomic inequalities in partial life expectancy for native-born and foreign-born populations 15](#_Toc221205685)

[**Figure S2** Differences in partial life expectancy between foreign-born and native-born populations in each country by occupation, stratified by gender and region of origin. *Note*: Partial life expectancy by occupational group is estimated for ages between 35 and 65. 16](#_Toc221205686)

[**Figure S3** Decomposition of the life expectancy gap between foreign-born and native-born populations in each country into the contributions of the mortality and educational structure of the population 17](#_Toc221205687)

# Section A: Data description

### Overview of harmonized data

| Country | Design | Coverage | Period | Missing data | | | | | |
| --- | --- | --- | --- | --- | --- | --- | --- | --- | --- |
|  |  |  |  | Country of birth | | Education | | Occupation | |
|  |  |  |  | PYs | Deaths | PYs | Deaths | PYs | Deaths |
| Northern Europe |  |  |  |  |  |  |  |  |  |
| Denmark | Longitudinal | All | 2015-2019 | 0.0% | 0.0% | 1.8% | 2.6% | 0.5% | 0.1% |
| Finland | Longitudinal | All | 2011-2018 | 0.0% | 0.0% | - | - | 0.1% | 0.0% |
| Sweden | Longitudinal | All | 2010-2017 | 0.0% | 0.0% | 1.1% | 1.6% | - | - |
| Western Europe |  |  |  |  |  |  |  |  |  |
| Austria | Longitudinal | All | 2013-2019 | 0.0% | 0.0% | 0.0% | 0.0% | 0.0% | 0.0% |
| Belgium | Longitudinal | All | 2011-2017 | 0.0% | 0.0% | 8.7% | 10.5% | - | - |
| Switzerland | Longitudinal | 20% sample | 2015-2018 | 0.3% | 0.3% | 0.0% | 0.0% | - | - |
| Southern Europe |  |  |  |  |  |  |  |  |  |
| Italy | Longitudinal | City (Turin) | 2011-2018 | 0.0% | 0.0% | 0.0% | 0.0% | - | - |
| Spain | Longitudinal | 9% sample | 2012-2019 | 0.0% | 0.0% | 0.0% | 0.0% | 0.0% | 0.0% |
| Eastern Europe |  |  |  |  |  |  |  |  |  |
| Estonia | Longitudinal | All | 2012-2019 | 0.0% | 0.0% | 0.2% | 0.6% | 1.0% | 0.2% |
| Lithuania | Longitudinal | All | 2011-2019 | 0.8% | 10.0% | 0.7% | 9.0% | 4.4% | 1.0% |

Abbreviation: PYs = person-years.

### Countries included in total, education-specific, and occupation-specific estimation of partial life expectancy for native-born and foreign-born populations

|  | AT | BE | CH | DK | EE | ES | FI | IT | LT | SE | Total |
| --- | --- | --- | --- | --- | --- | --- | --- | --- | --- | --- | --- |
| Total |  |  |  |  |  |  |  |  |  |  |  |
| Native-born | ● | ● | ● | ● | ● | ● | ● | ● | ● | ● | 10 countries |
| Foreign-born (total) | ● | ● | ● | ● | ● | ● | ● | ● | ● | ● | 10 countries |
| Foreign-born (European) | ● | ● ^b^ | ● | ● | ● | ● | × | ● | ● | ● | 9 countries |
| Foreign-born (non-European) | ● | ● | ● | ● | ● | ● | × | ● | ● | ● ^a^ | 9 countries |
| Education-specific |  |  |  |  |  |  |  |  |  |  |  |
| Native-born | ● | ● | ● | ● | ● | ● | × | ● | ● | ● | 9 countries |
| Foreign-born (total) | ● | ● | ● | ● | ● | ● | × | ● | ● | ● | 9 countries |
| Foreign-born (European) | ● | ● ^b^ | ● | ● | ● | ● | × | ● | ● | ● | 9 countries |
| Foreign-born (non-European) | ● | ● | ● | ● | × | ● | × | ● | × | ● | 7 countries |
| Occupation-specific |  |  |  |  |  |  |  |  |  |  |  |
| Native-born | ● | × | × | ● | ● | ● | ● | × | ● | × | 6 countries |
| Foreign-born (total) | ● | × | × | ● | ● | ● | ● | × | ● | × | 6 countries |
| Foreign-born (European) | ● | × ^b^ | × | ● | ● | ● | × | × | ● | × | 5 countries |
| Foreign-born (non-European) | ● | × | × | ● | × | ● | × | × | × | × | 3 countries |

^a^ For Sweden, the distinction between Western and non-Western among non-European foreign-born populations was not available due to data access restrictions.

^b^ For Belgium, Western non-European countries are aggregated with European countries.

Abbreviations: AT = Austria, BE = Belgium, DK = Denmark, EE = Estonia, FI = Finland, IT = Italy (Turin), LT = Lithuania, ES = Spain (9% sample), SE = Sweden, CH = Switzerland (20% sample).

### Distribution of person-years and deaths by country of birth status and education (%)

|  |  |  | Native-born | | | | Foreign-born  (total) | | | | Foreign-born  (European) | | | | Foreign-born  (non-European) | | | |
| --- | --- | --- | --- | --- | --- | --- | --- | --- | --- | --- | --- | --- | --- | --- | --- | --- | --- | --- |
|  |  |  | Low | Mid | High | NA | Low | Mid | High | NA | Low | Mid | High | NA | Low | Mid | High | NA |
| **Person-years** | | | | | | | | | | | | | | | | | | |
|  | Men | | | | | | | | | | | | | | | | | |
|  |  | Denmark | 22.2 | 47.8 | 28.8 | 1.2 | 22.1 | 34.5 | 33.1 | 10.2 | 19.2 | 37.6 | 33.1 | 10.0 | 26.0 | 30.5 | 33.0 | 10.5 |
|  |  | Finland | 25.6 | 42.6 | 31.8 | 0.0 | 50.4 | 26.9 | 22.7 | 0.0 | - | - | - | - | - | - | - | - |
|  |  | Sweden | 20.7 | 48.5 | 30.6 | 0.2 | 22.1 | 39.2 | 32.8 | 6.0 | 19.8 | 43.7 | 29.3 | 7.1 | 24.6 | 34.1 | 36.7 | 4.7 |
|  |  | Austria | 10.8 | 59.6 | 29.5 | 0.0 | 36.9 | 40.8 | 22.4 | 0.0 | 36.4 | 42.5 | 21.2 | 0.0 | 39.8 | 30.4 | 29.8 | 0.0 |
|  |  | Belgium | 35.7 | 32.7 | 27.2 | 4.4 | 33.6 | 16.4 | 18.0 | 32.0 | 32.4 | 16.7 | 16.4 | 34.5 | 34.8 | 16.1 | 19.5 | 29.6 |
|  |  | Switzerland | 8.6 | 51.2 | 40.2 | 0.0 | 33.3 | 31.6 | 35.0 | 0.0 | 33.9 | 32.9 | 33.3 | 0.0 | 30.9 | 25.9 | 43.2 | 0.0 |
|  |  | Italy | 45.9 | 34.9 | 19.2 | 0.0 | 44.9 | 40.9 | 14.2 | 0.0 | 34.7 | 52.4 | 12.9 | 0.0 | 53.3 | 31.3 | 15.3 | 0.0 |
|  |  | Spain | 58.4 | 24.6 | 17.0 | 0.0 | 42.8 | 34.4 | 22.8 | 0.0 | 39.6 | 37.4 | 23.0 | 0.0 | 45.3 | 32.2 | 22.6 | 0.0 |
|  |  | Estonia | 20.7 | 57.1 | 21.9 | 0.3 | 11.7 | 62.6 | 25.4 | 0.3 | 11.8 | 62.9 | 25.0 | 0.3 | 3.1 | 29.5 | 66.1 | 1.3 |
|  |  | Lithuania | 18.2 | 62.0 | 19.8 | 0.0 | 11.4 | 61.9 | 26.7 | 0.0 | 11.5 | 62.1 | 26.4 | 0.0 | 6.0 | 41.5 | 52.5 | 0.0 |
|  | Women | | | | | | | | | | | | | | | | | |
|  |  | Denmark | 23.5 | 39.8 | 35.9 | 0.8 | 25.1 | 32.9 | 33.7 | 8.3 | 20.1 | 33.3 | 38.6 | 7.9 | 31.3 | 32.3 | 27.5 | 8.8 |
|  |  | Finland | 22.7 | 37.9 | 39.4 | 0.0 | 41.2 | 27.6 | 31.2 | 0.0 | - | - | - | - | - | - | - | - |
|  |  | Sweden | 15.9 | 46.2 | 37.8 | 0.1 | 24.5 | 36.0 | 34.5 | 5.0 | 21.0 | 40.1 | 34.9 | 4.1 | 28.8 | 30.9 | 34.1 | 6.2 |
|  |  | Austria | 26.0 | 52.5 | 21.5 | 0.0 | 44.6 | 36.0 | 19.4 | 0.0 | 44.7 | 36.7 | 18.6 | 0.0 | 43.8 | 31.6 | 24.6 | 0.0 |
|  |  | Belgium | 36.2 | 30.5 | 29.4 | 3.8 | 36.0 | 15.9 | 17.8 | 30.3 | 35.7 | 17.5 | 17.6 | 29.2 | 36.2 | 14.3 | 17.9 | 31.5 |
|  |  | Switzerland | 19.0 | 59.4 | 21.6 | 0.0 | 38.2 | 32.1 | 29.7 | 0.0 | 38.6 | 33.3 | 28.1 | 0.0 | 36.9 | 28.0 | 35.1 | 0.0 |
|  |  | Italy | 49.4 | 32.5 | 18.1 | 0.0 | 42.7 | 40.8 | 16.6 | 0.0 | 31.8 | 50.5 | 17.7 | 0.0 | 55.2 | 29.6 | 15.2 | 0.0 |
|  |  | Spain | 59.5 | 20.9 | 19.6 | 0.0 | 42.7 | 34.0 | 23.3 | 0.0 | 38.5 | 37.3 | 24.1 | 0.0 | 45.5 | 31.7 | 22.7 | 0.0 |
|  |  | Estonia | 13.7 | 55.3 | 30.8 | 0.2 | 11.6 | 63.3 | 25.0 | 0.1 | 11.6 | 63.4 | 24.9 | 0.1 | 5.1 | 37.7 | 56.3 | 0.9 |
|  |  | Lithuania | 15.5 | 59.6 | 24.9 | 0.0 | 11.9 | 60.9 | 27.1 | 0.0 | 11.9 | 61.0 | 27.1 | 0.0 | 8.6 | 43.3 | 48.1 | 0.0 |
| **Deaths** | | | | | | | | | | | | | | | | | | |
|  | Men | | | | | | | | | | | | | | | | | |
|  |  | Denmark | 37.0 | 44.8 | 15.8 | 2.3 | 24.6 | 39.7 | 25.1 | 10.6 | 22.8 | 42.7 | 24.8 | 9.7 | 28.5 | 33.4 | 25.7 | 12.4 |
|  |  | Finland | 48.0 | 34.4 | 17.6 | 0.0 | 51.4 | 29.1 | 19.5 | 0.0 | 0.0 | 0.0 | 0.0 | 0.0 | 0.0 | 0.0 | 0.0 | 0.0 |
|  |  | Sweden | 40.3 | 42.1 | 16.8 | 0.7 | 34.9 | 40.5 | 18.0 | 6.6 | 35.8 | 42.9 | 15.2 | 6.0 | 31.9 | 32.1 | 27.3 | 8.6 |
|  |  | Austria | 22.1 | 61.6 | 16.2 | 0.0 | 39.7 | 42.9 | 17.4 | 0.0 | 39.9 | 43.8 | 16.3 | 0.0 | 37.0 | 32.0 | 31.0 | 0.0 |
|  |  | Belgium | 54.5 | 22.1 | 14.4 | 9.0 | 52.0 | 14.5 | 11.0 | 22.5 | 55.5 | 14.8 | 8.8 | 20.8 | 46.4 | 14.0 | 14.5 | 25.2 |
|  |  | Switzerland | 18.3 | 57.7 | 24.0 | 0.0 | 39.4 | 37.5 | 23.1 | 0.0 | 40.3 | 38.2 | 21.5 | 0.0 | 32.1 | 31.0 | 36.9 | 0.0 |
|  |  | Italy | 66.9 | 23.9 | 9.3 | 0.0 | 54.8 | 33.9 | 11.3 | 0.0 | 46.1 | 43.5 | 10.3 | 0.0 | 63.0 | 24.8 | 12.2 | 0.0 |
|  |  | Spain | 77.5 | 13.7 | 8.8 | 0.0 | 48.4 | 29.6 | 22.0 | 0.0 | 49.4 | 32.3 | 18.3 | 0.0 | 47.1 | 25.9 | 27.0 | 0.0 |
|  |  | Estonia | 39.6 | 47.7 | 12.1 | 0.7 | 25.8 | 57.7 | 16.1 | 0.4 | 25.8 | 57.8 | 16.1 | 0.4 | 13.3 | 46.7 | 40.0 | 0.0 |
|  |  | Lithuania | 36.1 | 53.2 | 10.7 | 0.0 | 23.0 | 59.0 | 18.0 | 0.0 | 23.0 | 59.1 | 17.9 | 0.0 | 15.0 | 30.0 | 55.0 | 0.0 |
|  | Women | | | | | | | | | | | | | | | | | |
|  |  | Denmark | 47.4 | 33.8 | 17.0 | 1.8 | 30.6 | 32.8 | 25.9 | 10.7 | 29.2 | 34.8 | 27.7 | 8.3 | 34.4 | 27.5 | 21.4 | 16.6 |
|  |  | Finland | 49.3 | 32.1 | 18.6 | 0.0 | 50.6 | 26.2 | 23.2 | 0.0 | 0.0 | 0.0 | 0.0 | 0.0 | 0.0 | 0.0 | 0.0 | 0.0 |
|  |  | Sweden | 36.9 | 43.7 | 18.8 | 0.6 | 38.2 | 35.7 | 17.5 | 8.6 | 38.7 | 38.7 | 16.5 | 6.0 | 35.6 | 22.9 | 21.8 | 19.7 |
|  |  | Austria | 46.0 | 45.1 | 9.0 | 0.0 | 52.2 | 35.8 | 12.0 | 0.0 | 52.8 | 35.9 | 11.3 | 0.0 | 44.4 | 33.9 | 21.7 | 0.0 |
|  |  | Belgium | 58.3 | 20.0 | 13.0 | 8.8 | 54.7 | 13.7 | 9.0 | 22.6 | 59.4 | 14.6 | 7.9 | 18.1 | 46.3 | 12.0 | 10.9 | 30.8 |
|  |  | Switzerland | 37.7 | 52.5 | 9.8 | 0.0 | 46.8 | 37.5 | 15.7 | 0.0 | 47.8 | 38.6 | 13.6 | 0.0 | 38.9 | 29.5 | 31.6 | 0.0 |
|  |  | Italy | 72.7 | 20.2 | 7.1 | 0.0 | 59.5 | 29.9 | 10.6 | 0.0 | 49.5 | 35.8 | 14.7 | 0.0 | 70.2 | 23.5 | 6.3 | 0.0 |
|  |  | Spain | 82.4 | 9.7 | 7.9 | 0.0 | 52.4 | 29.0 | 18.6 | 0.0 | 51.6 | 30.7 | 17.8 | 0.0 | 53.3 | 27.1 | 19.6 | 0.0 |
|  |  | Estonia | 32.6 | 51.8 | 14.9 | 0.7 | 28.0 | 56.0 | 15.4 | 0.6 | 28.0 | 56.1 | 15.4 | 0.6 | 27.3 | 27.3 | 45.5 | 0.0 |
|  |  | Lithuania | 39.8 | 47.9 | 12.3 | 0.0 | 29.1 | 54.9 | 16.1 | 0.0 | 29.1 | 54.8 | 16.1 | 0.0 | 0.0 | 80.0 | 20.0 | 0.0 |

Abbreviation: NA = not available.

### Definition of subgroups based on the country of birth

| Subgroups | Country of birth |
| --- | --- |
| Native-born | Country of residence |
| Foreign-born |  |
| Born in European countries | Aland, Albania, Andorra, Armenia, Austria, Azerbaijan, Belarus, Belgium, Bosnia and Herzegovina, Bulgaria, Croatia, Cyprus, Czech Republic, Denmark, Estonia, Faroe Islands, Finland, France, Georgia, Germany, Gibraltar, Greece, Greenland, Guernsey, Hungary, Iceland, Ireland, Isle of Man, Italy, Jersey, Kazakhstan, Kosovo, Kyrgyzstan, Latvia, Liechtenstein, Lithuania, Luxembourg, Malta, Moldova, Monaco, Montenegro, Netherlands, North Macedonia, Norway, Poland, Portugal, Romania, Russia, San Marino, Serbia, Slovakia, Slovenia, Spain, Sweden, Switzerland, Tajikistan, Turkey, Turkmenistan, Ukraine, United Kingdom, Uzbekistan, Vatican City |
| Born in non-European countries |  |
| Born in Western countries ^a^ | United States of America, New Zealand, Australia, Canada |
| Born in non-Western countries | All other countries |

^a^ For Belgium, Western non-European countries are aggregated with European countries.

### Collection of educational information among foreign-born populations in each country

| Country | Primary source of data | Supplementary data | Missing education |
| --- | --- | --- | --- |
| Denmark | Administrative registers that are created and used at Danish educational institutions | - Population and Housing Census (1970) - Validation of a foreign education by Danish authorities - Surveys on the education brought by immigrants carried out by Statistics Denmark | Classified as “missing” |
| Finland ^a^ | Finnish educational establishments on educational certificates obtained in Finland | - No additional data | Classified as “low education” |
| Sweden | Swedish educational registers on educational certificates obtained in Sweden | - Validation of a foreign education by Swedish authorities - Records from the employment agency - The survey of foreign-born, which has been mailed to all immigrants aged 20–59 whose educational information is missing in the year after arrival (since 1999) | Classified as “missing” |
| Austria | Austrian administrative registers | - No additional data | Imputed using logistic regression models |
| Belgium | Self-reported census in 2001 for everyone above age 15 | - No additional data | Classified as “missing” |
| Switzerland | Self-reported census | - Educational information is checked for plausibility and is modified using information on learned and practiced profession/occupation, where applicable | Classified as “low education” |
| Italy (Turin) | Self-reported census | - Census information has been updated by the municipal population registers | Classified as “missing” |
| Spain | Self-reported census | - No additional data | Imputed using statistical regression models |
| Estonia | Self-reported census | - Census information has been updated by the Estonian Education System since 2004 | Classified as “missing” |
| Lithuania | Self-reported census | - No additional data | Classified as “missing” |

^a^ Excluded from the education-specific analysis

# Section B: Mortality smoothing

## Methods

One of the most common challenges faced by researchers dealing with mortality is that the death counts at older ages often show fluctuating patterns from one age point to another due to the low number of person-year exposures.^1^ Our data on the mortality of foreign-born populations, too, showed highly unstable patterns in several countries at older ages due to the low numbers of population exposure counts. The fluctuation was particularly pronounced when stratified by education and detailed country of birth (by region, i.e., European versus non-European origins).

A common method used to overcome this issue is the penalized *B*-splines, i.e., *P*-splines.^1,2^ *P*-splines enable the flexible and accurate fit of the data. Notably, death counts of the foreign-born group can be under the influence of some selectivity issues, such as the healthy immigrant effect and the return migration bias.^3^ Because of the potential selection issue, the age-related changes in the mortality of foreign-born populations may differ from those of their native-born counterparts. Therefore, it is important to allow for flexibility in the age-related mortality patterns while ensuring accuracy when smoothing mortality rates by country of birth.

To compare our main results using the observed mortality by country of birth, we estimated smoothed mortality rates using generalized additive models. We include *B*-splines for the age variable and the interaction term between the age and the country of birth to ensure flexibility in the age-related mortality patterns of foreign-born populations. We calculated the smoothed mortality for each subgroup stratified by country of birth groups (native- vs. foreign-born), gender, and educational attainment in each receiving country.

## Results


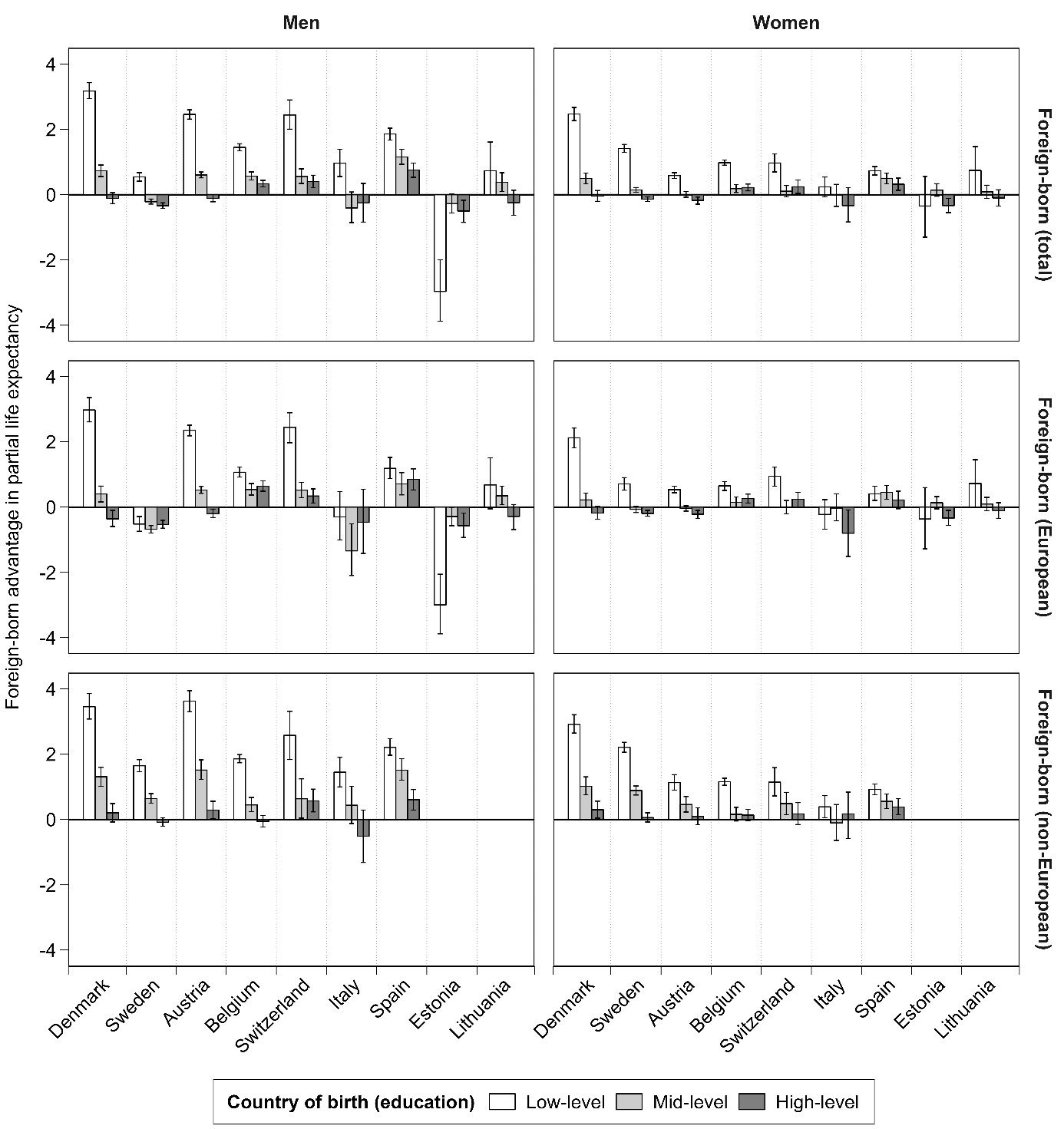


#### Differences in partial life expectancy between foreign-born and native-born populations in each country by education in samples stratified by gender and region of origin, estimated from the smoothed mortality

## References

1. Eilers PHC, Marx BD. Flexible smoothing with B-splines and penalties. *Stat Sci*. Institute of Mathematical Statistics; 1996 May 1;**11**(2):89–121.

2. Currie ID, Durban M, Eilers PH. Smoothing and forecasting mortality rates. *Stat Modelling*. Sage PublicationsSage CA: Thousand Oaks, CA; 2004 Dec 1;**4**(4):279–298.

3. Abraído-Lanza AF, Dohrenwend BP, Ng-Mak DS, Turner JB. The Latino mortality paradox: a test of the ‘salmon bias’ and healthy migrant hypotheses. *Am J Public Health*. American Public Health Association; 1999 Oct 7;**89**(10):1543–1548.

# Section C: Supplemental findings

### Partial life expectancy gap between native-born and foreign-born populations from non-European countries (total non-European versus non-Western non-European)

|  |  | Native-born | Foreign-born (non-European, total) | | Foreign-born (non-European, non-Western) | |
| --- | --- | --- | --- | --- | --- | --- |
|  |  | PLE ^a^ | PLE ^a^ | Gap ^b^ | PLE ^a^ | Gap ^b^ |
| Men |  |  |  |  |  |  |
|  | Europe (mean) | 40.6 | 42.0 | 1.4 ( 1.4, 1.4) | 41.9 | 1.3 ( 1.3, 1.3) |
|  | Denmark | 40.2 | 41.8 | 1.6 ( 1.4, 1.7) | 41.8 | 1.5 ( 1.4, 1.7) |
|  | Finland | 39.7 | - | - | - | - |
|  | Sweden | 41.2 | 41.9 | 0.6 ( 0.6, 0.7) | - | - |
|  | Austria | 40.1 | 41.5 | 1.4 ( 1.3, 1.6) | 41.5 | 1.4 ( 1.3, 1.6) |
|  | Belgium | 39.7 | 40.8 | 1.1 ( 1.0, 1.2) | 40.2 | 0.5 ( 0.3, 0.7) |
|  | Switzerland | 41.6 | 42.5 | 1.0 ( 0.7, 1.2) | 42.5 | 1.0 ( 0.7, 1.3) |
|  | Italy | 41.2 | 42.0 | 0.9 ( 0.6, 1.2) | 42.0 | 0.8 ( 0.5, 1.2) |
|  | Spain | 40.4 | 42.3 | 1.8 ( 1.7, 2.0) | 42.3 | 1.8 ( 1.7, 2.0) |
|  | Estonia | 36.6 | 42.2 | 5.6 ( 4.2, 6.8) | 41.8 | 5.2 ( 3.3, 6.8) |
|  | Lithuania | 35.7 | 41.6 | 5.9 ( 4.5, 7.4) | 40.6 | 5.0 ( 3.1, 6.9) |
| Women |  |  |  |  |  |  |
|  | Europe (mean) | 42.5 | 43.1 | 0.6 ( 0.6, 0.6) | 43.0 | 0.6 ( 0.6, 0.6) |
|  | Denmark | 41.8 | 43.0 | 1.2 ( 1.1, 1.4) | 43.0 | 1.2 ( 1.1, 1.4) |
|  | Finland | 42.3 | - | - | - | - |
|  | Sweden | 42.4 | 43.0 | 0.6 ( 0.6, 0.7) | - | - |
|  | Austria | 42.2 | 42.8 | 0.6 ( 0.5, 0.7) | 42.8 | 0.6 ( 0.4, 0.7) |
|  | Belgium | 41.8 | 42.4 | 0.6 ( 0.6, 0.7) | 42.3 | 0.5 ( 0.4, 0.7) |
|  | Switzerland | 42.9 | 43.4 | 0.6 ( 0.4, 0.8) | 43.5 | 0.6 ( 0.4, 0.8) |
|  | Italy | 42.6 | 42.9 | 0.3 ( 0.0, 0.5) | 42.9 | 0.3 ( 0.0, 0.5) |
|  | Spain | 42.7 | 43.4 | 0.7 ( 0.6, 0.8) | 43.4 | 0.7 ( 0.6, 0.8) |
|  | Estonia | 41.5 | 42.8 | 1.4 ( 0.1, 2.6) | 43.4 | 2.0 ( 0.7, 3.0) |
|  | Lithuania | 41.0 | 42.0 | 1.0 (-0.9, 2.8) | 41.1 | 0.1 (-2.6, 2.5) |

^a^ Partial life expectancy between ages 35 and 80

^b^ Foreign-born differential in partial life expectancy compared to native-born populations

### Partial life expectancy gap between native-born and foreign-born populations by socioeconomic status

|  |  |  | Native-born | Foreign-born (total) | | Foreign-born (European) | | Foreign-born (non-European) | |
| --- | --- | --- | --- | --- | --- | --- | --- | --- | --- |
|  |  |  | PLE ^a^ | PLE ^a^ | Gap ^b^ | PLE ^a^ | Gap ^b^ | PLE ^a^ | Gap ^b^ |
| Men | | | | | | | | | |
|  | Low-level education | | | | | | | | |
|  |  | Europe (mean) | 39.3 | 41.0 | 1.6 ( 1.6,  1.6) | 40.3 | 1.0 ( 1.0,  1.0) | 41.7 | 2.3 ( 2.3,  2.3) |
|  |  | Denmark | 37.7 | 40.9 | 3.2 ( 3.0,  3.5) | 40.7 | 3.0 ( 2.7,  3.4) | 41.2 | 3.5 ( 3.2,  3.9) |
|  |  | Sweden | 39.6 | 40.2 | 0.6 ( 0.5,  0.8) | 39.2 | -0.3 (-0.6, -0.1) | 41.3 | 1.7 ( 1.5,  1.9) |
|  |  | Austria | 37.4 | 39.8 | 2.4 ( 2.3,  2.5) | 39.7 | 2.3 ( 2.2,  2.4) | 41.0 | 3.6 ( 3.3,  4.0) |
|  |  | Belgium | 38.6 | 40.0 | 1.5 ( 1.4,  1.6) | 39.6 | 1.0 ( 0.9,  1.2) | 40.5 | 1.9 ( 1.8,  2.1) |
|  |  | Switzerland | 39.3 | 41.7 | 2.4 ( 2.0,  2.9) | 41.7 | 2.5 ( 2.0,  2.9) | 41.9 | 2.7 ( 2.0,  3.4) |
|  |  | Italy | 40.3 | 41.4 | 1.1 ( 0.7,  1.5) | 40.1 | -0.2 (-1.0,  0.6) | 41.9 | 1.6 ( 1.2,  2.0) |
|  |  | Spain | 39.9 | 41.7 | 1.9 ( 1.7,  2.0) | 41.2 | 1.3 ( 1.0,  1.6) | 42.1 | 2.2 ( 1.9,  2.5) |
|  |  | Estonia | 33.3 | 30.7 | -2.6 (-3.5, -1.6) | 30.6 | -2.7 (-3.6, -1.6) | - | - |
|  |  | Lithuania | 31.9 | 32.5 | 0.6 (-0.2,  1.4) | 32.4 | 0.5 (-0.3,  1.3) | - | - |
|  | Mid-level education | | | | | | | | |
|  |  | Europe (mean) | 40.9 | 41.3 | 0.4 ( 0.4,  0.5) | 40.9 | 0.0 ( 0.0,  0.0) | 42.0 | 1.1 ( 1.0,  1.1) |
|  |  | Denmark | 40.5 | 41.3 | 0.7 ( 0.6,  0.9) | 40.9 | 0.4 ( 0.2,  0.7) | 41.8 | 1.3 ( 1.0,  1.6) |
|  |  | Sweden | 41.2 | 40.9 | -0.2 (-0.3, -0.1) | 40.5 | -0.7 (-0.8, -0.5) | 41.8 | 0.7 ( 0.5,  0.8) |
|  |  | Austria | 39.8 | 40.4 | 0.6 ( 0.5,  0.7) | 40.3 | 0.5 ( 0.4,  0.6) | 41.4 | 1.5 ( 1.3,  1.8) |
|  |  | Belgium | 40.0 | 40.6 | 0.6 ( 0.4,  0.7) | 40.6 | 0.5 ( 0.4,  0.7) | 40.5 | 0.5 ( 0.3,  0.7) |
|  |  | Switzerland | 41.2 | 41.8 | 0.5 ( 0.3,  0.8) | 41.7 | 0.5 ( 0.3,  0.8) | 42.0 | 0.8 ( 0.2,  1.4) |
|  |  | Italy | 41.7 | 41.5 | -0.2 (-0.7,  0.2) | 40.7 | -1.0 (-1.8, -0.2) | 42.2 | 0.5 (-0.1,  1.1) |
|  |  | Spain | 40.9 | 42.0 | 1.1 ( 0.9,  1.3) | 41.6 | 0.7 ( 0.4,  1.0) | 42.3 | 1.5 ( 1.2,  1.8) |
|  |  | Estonia | 36.8 | 36.4 | -0.4 (-0.7, -0.1) | 36.4 | -0.4 (-0.7, -0.1) | - | - |
|  |  | Lithuania | 35.6 | 36.0 | 0.4 ( 0.1,  0.7) | 36.0 | 0.4 ( 0.1,  0.6) | - | - |
|  | High-level education | | | | | | | | |
|  |  | Europe (mean) | 42.0 | 42.3 | 0.2 ( 0.2,  0.3) | 42.3 | 0.3 ( 0.2,  0.3) | 42.4 | 0.3 ( 0.3,  0.3) |
|  |  | Denmark | 42.1 | 42.0 | -0.1 (-0.3,  0.1) | 41.8 | -0.3 (-0.6, -0.1) | 42.4 | 0.2 (-0.1,  0.5) |
|  |  | Sweden | 42.5 | 42.2 | -0.3 (-0.4, -0.3) | 42.0 | -0.5 (-0.6, -0.4) | 42.4 | -0.1 (-0.2,  0.1) |
|  |  | Austria | 41.8 | 41.7 | -0.1 (-0.2,  0.0) | 41.6 | -0.2 (-0.3, -0.1) | 42.2 | 0.3 ( 0.1,  0.6) |
|  |  | Belgium | 41.6 | 41.9 | 0.3 ( 0.2,  0.5) | 42.2 | 0.6 ( 0.5,  0.8) | 41.5 | 0.0 (-0.2,  0.2) |
|  |  | Switzerland | 42.5 | 43.0 | 0.4 ( 0.2,  0.6) | 42.9 | 0.3 ( 0.1,  0.5) | 43.2 | 0.7 ( 0.3,  1.0) |
|  |  | Italy | 42.4 | 42.3 | -0.2 (-0.8,  0.4) | 42.3 | -0.2 (-1.1,  0.7) | 42.3 | -0.2 (-1.0,  0.6) |
|  |  | Spain | 41.8 | 42.6 | 0.8 ( 0.6,  1.0) | 42.6 | 0.8 ( 0.5,  1.2) | 42.5 | 0.7 ( 0.4,  1.0) |
|  |  | Estonia | 40.3 | 39.8 | -0.4 (-0.7, -0.1) | 39.8 | -0.5 (-0.8, -0.1) | - | - |
|  |  | Lithuania | 39.8 | 39.5 | -0.3 (-0.7,  0.1) | 39.4 | -0.4 (-0.8,  0.0) | - | - |
|  | Manual occupation | | | | | | | | |
|  |  | Europe (mean) | 28.9 | 29.3 | 0.4 ( 0.4,  0.4) | 29.2 | 0.2 ( 0.2,  0.2) | 29.6 | 0.6 ( 0.6,  0.6) |
|  |  | Denmark | 29.4 | 29.6 | 0.2 ( 0.1,  0.2) | 29.5 | 0.1 ( 0.1,  0.2) | 29.7 | 0.3 ( 0.2,  0.4) |
|  |  | Finland | 28.3 | 29.0 | 0.7 ( 0.6,  0.8) | - | - | - | - |
|  |  | Austria | 29.1 | 29.4 | 0.3 ( 0.3,  0.3) | 29.4 | 0.3 ( 0.2,  0.3) | 29.5 | 0.4 ( 0.3,  0.5) |
|  |  | Spain | 29.0 | 29.4 | 0.3 ( 0.3,  0.4) | 29.1 | 0.1 (-0.1,  0.2) | 29.6 | 0.5 ( 0.4,  0.6) |
|  |  | Estonia | 28.4 | 28.3 | -0.1 (-0.3,  0.1) | 28.3 | -0.1 (-0.3,  0.2) | - | - |
|  |  | Lithuania | 27.4 | 27.7 | 0.3 ( 0.1,  0.5) | 27.7 | 0.3 ( 0.0,  0.5) | - | - |
|  | Non-manual occupation | | | | | | | | |
|  |  | Europe (mean) | 29.3 | 29.5 | 0.1 ( 0.1,  0.1) | 29.5 | 0.1 ( 0.1,  0.1) | 29.5 | 0.2 ( 0.2,  0.2) |
|  |  | Denmark | 29.6 | 29.7 | 0.1 ( 0.0,  0.2) | 29.6 | 0.1 ( 0.0,  0.1) | 29.7 | 0.2 ( 0.1,  0.2) |
|  |  | Finland | 29.2 | 29.3 | 0.2 ( 0.1,  0.2) | - | - | - | - |
|  |  | Austria | 29.5 | 29.5 | 0.1 ( 0.0,  0.1) | 29.5 | 0.0 ( 0.0,  0.1) | 29.6 | 0.1 ( 0.0,  0.2) |
|  |  | Spain | 29.4 | 29.5 | 0.1 ( 0.0,  0.2) | 29.5 | 0.1 ( 0.0,  0.3) | 29.5 | 0.1 ( 0.0,  0.3) |
|  |  | Estonia | 29.1 | 29.1 | 0.0 (-0.1,  0.2) | 29.1 | 0.0 (-0.1,  0.2) | - | - |
|  |  | Lithuania | 28.7 | 28.6 | -0.1 (-0.4,  0.1) | 28.5 | -0.2 (-0.4,  0.1) | - | - |
|  | Other occupation | | | | | | | | |
|  |  | Europe (mean) | 28.9 | 29.6 | 0.6 ( 0.6,  0.6) | 29.4 | 0.5 ( 0.5,  0.5) | 29.7 | 0.8 ( 0.8,  0.8) |
|  |  | Denmark | 29.6 | 29.7 | 0.1 ( 0.0,  0.2) | 29.6 | 0.1 (-0.1,  0.2) | 29.7 | 0.2 ( 0.0,  0.3) |
|  |  | Finland | 28.9 | 29.3 | 0.4 ( 0.3,  0.5) | - | - | - | - |
|  |  | Austria | 29.3 | 29.4 | 0.1 ( 0.1,  0.2) | 29.4 | 0.1 ( 0.0,  0.2) | 29.5 | 0.2 ( 0.0,  0.3) |
|  |  | Spain | 28.8 | 29.6 | 0.8 ( 0.6,  1.0) | 29.4 | 0.5 ( 0.2,  0.9) | 29.8 | 0.9 ( 0.7,  1.1) |
|  |  | Estonia | 28.6 | 28.1 | -0.5 (-3.8,  1.4) | 28.0 | -0.6 (-4.0,  1.4) | - | - |
|  |  | Lithuania | 28.4 | 28.8 | 0.4 ( 0.1,  0.6) | 28.8 | 0.3 ( 0.1,  0.6) | - | - |
| Women | | | | | | | | | |
|  | Low-level education | | | | | | | | |
|  |  | Europe (mean) | 41.8 | 42.6 | 0.8 ( 0.8,  0.8) | 42.3 | 0.5 ( 0.5,  0.5) | 43.0 | 1.2 ( 1.2,  1.2) |
|  |  | Denmark | 39.8 | 42.4 | 2.6 ( 2.4,  2.8) | 42.0 | 2.2 ( 1.9,  2.5) | 42.8 | 3.1 ( 2.8,  3.4) |
|  |  | Sweden | 40.6 | 42.1 | 1.5 ( 1.3,  1.6) | 41.4 | 0.8 ( 0.6,  1.0) | 42.9 | 2.3 ( 2.1,  2.4) |
|  |  | Austria | 41.3 | 42.0 | 0.6 ( 0.5,  0.7) | 41.9 | 0.6 ( 0.5,  0.7) | 42.6 | 1.3 ( 1.0,  1.5) |
|  |  | Belgium | 41.0 | 42.0 | 1.0 ( 0.9,  1.1) | 41.6 | 0.6 ( 0.5,  0.8) | 42.2 | 1.2 ( 1.1,  1.3) |
|  |  | Switzerland | 41.9 | 43.0 | 1.1 ( 0.8,  1.4) | 43.0 | 1.1 ( 0.7,  1.4) | 43.2 | 1.3 ( 0.8,  1.7) |
|  |  | Italy | 42.3 | 42.6 | 0.4 ( 0.1,  0.7) | 42.4 | 0.1 (-0.4,  0.5) | 42.8 | 0.6 ( 0.2,  0.9) |
|  |  | Spain | 42.5 | 43.2 | 0.8 ( 0.6,  0.9) | 42.9 | 0.4 ( 0.2,  0.6) | 43.4 | 0.9 ( 0.8,  1.1) |
|  |  | Estonia | 38.9 | 38.3 | -0.6 (-1.6,  0.4) | 38.3 | -0.6 (-1.6,  0.4) | - | - |
|  |  | Lithuania | 38.1 | 38.9 | 0.9 ( 0.1,  1.7) | 38.9 | 0.9 ( 0.0,  1.6) | - | - |
|  | Mid-level education | | | | | | | | |
|  |  | Europe (mean) | 42.6 | 42.8 | 0.2 ( 0.2,  0.2) | 42.8 | 0.2 ( 0.1,  0.2) | 43.0 | 0.4 ( 0.4,  0.4) |
|  |  | Denmark | 42.1 | 42.6 | 0.5 ( 0.3,  0.7) | 42.3 | 0.2 ( 0.0,  0.4) | 43.1 | 1.0 ( 0.8,  1.3) |
|  |  | Sweden | 42.3 | 42.5 | 0.1 ( 0.1,  0.2) | 42.3 | -0.1 (-0.2,  0.0) | 43.2 | 0.9 ( 0.8,  1.1) |
|  |  | Austria | 42.3 | 42.3 | 0.0 (-0.1,  0.1) | 42.3 | 0.0 (-0.1,  0.0) | 42.9 | 0.5 ( 0.3,  0.8) |
|  |  | Belgium | 42.0 | 42.2 | 0.2 ( 0.1,  0.3) | 42.1 | 0.1 ( 0.0,  0.3) | 42.1 | 0.1 (-0.1,  0.3) |
|  |  | Switzerland | 43.0 | 43.1 | 0.1 (-0.1,  0.3) | 43.0 | 0.0 (-0.2,  0.2) | 43.5 | 0.6 ( 0.2,  0.9) |
|  |  | Italy | 42.8 | 42.8 | 0.0 (-0.4,  0.3) | 42.8 | 0.0 (-0.4,  0.5) | 42.8 | -0.1 (-0.6,  0.5) |
|  |  | Spain | 42.9 | 43.4 | 0.5 ( 0.3,  0.6) | 43.3 | 0.4 ( 0.2,  0.7) | 43.4 | 0.5 ( 0.3,  0.8) |
|  |  | Estonia | 41.4 | 41.5 | 0.1 (-0.1,  0.2) | 41.5 | 0.0 (-0.2,  0.2) | - | - |
|  |  | Lithuania | 41.0 | 41.1 | 0.1 (-0.1,  0.3) | 41.1 | 0.1 (-0.1,  0.3) | - | - |
|  | High-level education | | | | | | | | |
|  |  | Europe (mean) | 43.1 | 43.1 | 0.1 ( 0.1,  0.1) | 43.0 | -0.1 (-0.1, -0.1) | 43.4 | 0.4 ( 0.3,  0.4) |
|  |  | Denmark | 42.9 | 42.8 | 0.0 (-0.2,  0.1) | 42.7 | -0.2 (-0.4,  0.0) | 43.3 | 0.4 ( 0.1,  0.6) |
|  |  | Sweden | 43.2 | 43.1 | -0.1 (-0.2, -0.1) | 43.0 | -0.2 (-0.3, -0.1) | 43.3 | 0.1 (-0.1,  0.2) |
|  |  | Austria | 43.0 | 42.8 | -0.2 (-0.3, -0.1) | 42.8 | -0.2 (-0.4, -0.1) | 43.1 | 0.1 (-0.2,  0.4) |
|  |  | Belgium | 42.7 | 43.0 | 0.2 ( 0.1,  0.3) | 43.0 | 0.3 ( 0.2,  0.4) | 42.9 | 0.1 ( 0.0,  0.3) |
|  |  | Switzerland | 43.3 | 43.6 | 0.3 ( 0.1,  0.5) | 43.6 | 0.3 ( 0.0,  0.5) | 43.6 | 0.3 (-0.1,  0.6) |
|  |  | Italy | 43.2 | 43.0 | -0.2 (-0.7,  0.3) | 42.6 | -0.6 (-1.3,  0.1) | 43.6 | 0.5 (-0.2,  1.1) |
|  |  | Spain | 43.1 | 43.4 | 0.3 ( 0.1,  0.5) | 43.4 | 0.3 ( 0.0,  0.5) | 43.5 | 0.4 ( 0.1,  0.7) |
|  |  | Estonia | 42.8 | 42.4 | -0.4 (-0.6, -0.1) | 42.4 | -0.4 (-0.6, -0.1) | - | - |
|  |  | Lithuania | 42.4 | 42.3 | -0.1 (-0.3,  0.1) | 42.3 | -0.1 (-0.3,  0.1) | - | - |
|  | Manual occupation | | | | | | | | |
|  |  | Europe (mean) | 29.5 | 29.7 | 0.2 ( 0.2,  0.2) | 29.6 | 0.1 ( 0.1,  0.1) | 29.7 | 0.3 ( 0.2,  0.3) |
|  |  | Denmark | 29.6 | 29.7 | 0.1 ( 0.1,  0.2) | 29.7 | 0.1 ( 0.0,  0.2) | 29.8 | 0.2 ( 0.1,  0.3) |
|  |  | Finland | 29.0 | 29.3 | 0.3 ( 0.2,  0.4) | - | - | - | - |
|  |  | Austria | 29.6 | 29.7 | 0.1 ( 0.1,  0.2) | 29.7 | 0.1 ( 0.1,  0.2) | 29.7 | 0.1 ( 0.0,  0.2) |
|  |  | Spain | 29.5 | 29.7 | 0.1 ( 0.1,  0.2) | 29.6 | 0.1 (-0.1,  0.2) | 29.7 | 0.2 ( 0.1,  0.3) |
|  |  | Estonia | 29.3 | 29.4 | 0.1 (-0.1,  0.3) | 29.4 | 0.1 (-0.1,  0.3) | - | - |
|  |  | Lithuania | 29.0 | 29.2 | 0.2 ( 0.0,  0.3) | 29.2 | 0.2 ( 0.0,  0.3) | - | - |
|  | Non-manual occupation | | | | | | | | |
|  |  | Europe (mean) | 29.6 | 29.7 | 0.1 ( 0.1,  0.1) | 29.7 | 0.1 ( 0.1,  0.1) | 29.7 | 0.1 ( 0.1,  0.1) |
|  |  | Denmark | 29.7 | 29.7 | 0.1 ( 0.0,  0.1) | 29.7 | 0.1 ( 0.0,  0.1) | 29.8 | 0.1 ( 0.0,  0.2) |
|  |  | Finland | 29.5 | 29.6 | 0.0 ( 0.0,  0.1) | - | - | - | - |
|  |  | Austria | 29.7 | 29.7 | 0.0 ( 0.0,  0.0) | 29.7 | 0.0 ( 0.0,  0.0) | 29.8 | 0.0 ( 0.0,  0.1) |
|  |  | Spain | 29.6 | 29.7 | 0.1 ( 0.0,  0.2) | 29.7 | 0.1 ( 0.0,  0.2) | 29.7 | 0.1 ( 0.0,  0.2) |
|  |  | Estonia | 29.5 | 29.5 | 0.0 (-0.1,  0.1) | 29.5 | 0.0 (-0.1,  0.1) | - | - |
|  |  | Lithuania | 29.4 | 29.4 | 0.0 (-0.1,  0.1) | 29.4 | 0.0 (-0.1,  0.1) | - | - |
|  | Other occupation | | | | | | | | |
|  |  | Europe (mean) | 29.6 | 29.7 | 0.1 ( 0.1,  0.1) | 29.8 | 0.2 ( 0.2,  0.2) | 29.6 | 0.1 ( 0.0,  0.1) |
|  |  | Denmark | 29.7 | 29.6 | -0.1 (-0.3,  0.0) | 29.6 | -0.1 (-0.3,  0.1) | 29.6 | -0.1 (-0.4,  0.1) |
|  |  | Finland | 29.4 | 29.5 | 0.1 ( 0.0,  0.3) | - | - | - | - |
|  |  | Austria | 29.6 | 29.6 | 0.0 (-0.1,  0.0) | 29.6 | 0.0 (-0.1,  0.0) | 29.7 | 0.0 (-0.1,  0.2) |
|  |  | Spain | 29.6 | 29.7 | 0.1 (-0.1,  0.4) | 29.8 | 0.3 ( 0.0,  0.5) | 29.6 | 0.0 (-0.4,  0.4) |
|  |  | Estonia | 29.4 | 29.8 | 0.3 ( 0.0,  0.7) | 29.8 | 0.3 ( 0.0,  0.7) | - | - |
|  |  | Lithuania | 29.3 | 29.3 | 0.0 (-0.4,  0.3) | 29.3 | 0.0 (-0.4,  0.3) | - | - |

^a^ Partial life expectancy between ages 35 and 80

^b^ Foreign-born differential in partial life expectancy compared to native-born populations

### Socioeconomic inequalities in partial life expectancy for native-born and foreign-born populations

|  |  |  | Native-born | Foreign-born (total) | Foreign-born (European) | Foreign-born (non-European) |
| --- | --- | --- | --- | --- | --- | --- |
| Educational inequalities ^a^ | | | | | | |
|  | Men | | | | | |
|  |  | Europe (mean) | 2.7 (  2.7,   2.7) | 1.3 (  1.3.   1.3) | 2.0 (  2.0.   2.0) | 0.7 (  0.7,   0.7) |
|  |  | Denmark | 4.5 (  4.4,   4.6) | 1.2 (  0.9.   1.5) | 1.1 (  0.7.   1.6) | 1.2 (  0.7,   1.6) |
|  |  | Finland | 4.6 (  4.5,   4.7) | 0.7 (  0.4.   1.1) | - | - |
|  |  | Sweden | 3.0 (  2.9,   3.0) | 2.0 (  1.9.   2.1) | 2.8 (  2.5.   3.0) | 1.2 (  1.0,   1.4) |
|  |  | Austria | 4.4 (  4.3,   4.5) | 1.9 (  1.7.   2.0) | 1.9 (  1.7.   2.0) | 1.1 (  0.8,   1.5) |
|  |  | Belgium | 3.0 (  2.9,   3.1) | 1.9 (  1.7.   2.0) | 2.6 (  2.4.   2.8) | 1.1 (  0.8,   1.3) |
|  |  | Switzerland | 3.3 (  2.8,   3.7) | 1.2 (  1.0.   1.5) | 1.1 (  0.9.   1.4) | 1.3 (  0.5,   1.9) |
|  |  | Italy | 2.1 (  1.9,   2.3) | 0.9 (  0.2.   1.6) | 2.1 (  0.9.   3.3) | 0.4 (- 0.5,   1.3) |
|  |  | Spain | 1.9 (  1.8,   1.9) | 0.8 (  0.5.   1.1) | 1.4 (  1.0.   1.9) | 0.4 (  0.0,   0.8) |
|  |  | Estonia | 7.0 (  6.7,   7.2) | 9.2 (  8.2,  10.1) | 9.1 (  8.2,  10.1) | - |
|  |  | Lithuania | 7.9 (  7.8,   8.0) | 7.1 (  6.1.   7.9) | 7.0 (  6.2.   7.9) | - |
|  | Women | | | | | |
|  |  | Europe (mean) | 1.3 (  1.3,   1.3) | 0.6 (  0.5.   0.6) | 0.7 (  0.7.   0.7) | 0.5 (  0.5,   0.5) |
|  |  | Denmark | 3.1 (  3.0,   3.2) | 0.5 (  0.3.   0.7) | 0.7 (  0.4.   1.0) | 0.4 (  0.0,   0.8) |
|  |  | Finland | 2.9 (  2.8,   3.0) | 0.6 (  0.4.   0.9) | - | - |
|  |  | Sweden | 2.6 (  2.5,   2.7) | 1.0 (  0.9.   1.1) | 1.6 (  1.4.   1.8) | 0.4 (  0.2,   0.6) |
|  |  | Austria | 1.6 (  1.6,   1.7) | 0.8 (  0.7.   1.0) | 0.8 (  0.7.   1.0) | 0.5 (  0.2,   0.8) |
|  |  | Belgium | 1.7 (  1.7,   1.8) | 1.0 (  0.9.   1.1) | 1.4 (  1.2.   1.6) | 0.7 (  0.5,   0.9) |
|  |  | Switzerland | 1.4 (  1.1,   1.7) | 0.6 (  0.4.   0.8) | 0.6 (  0.4.   0.8) | 0.4 (- 0.1,   0.9) |
|  |  | Italy | 0.9 (  0.7,   1.1) | 0.3 (- 0.2.   0.9) | 0.2 (- 0.6.   1.0) | 0.8 (  0.1,   1.5) |
|  |  | Spain | 0.6 (  0.6,   0.7) | 0.2 (  0.0.   0.4) | 0.5 (  0.1.   0.8) | 0.1 (- 0.2,   0.4) |
|  |  | Estonia | 3.9 (  3.6,   4.1) | 4.1 (  3.1.   5.2) | 4.1 (  3.1.   5.2) | - |
|  |  | Lithuania | 4.4 (  4.2,   4.5) | 3.4 (  2.7.   4.2) | 3.4 (  2.7.   4.2) | - |
| Occupational inequalities ^b^ | | | | | | |
|  | Men | | | | | |
|  |  | Europe (mean) | 0.4 (  0.4,   0.4) | 0.1 (  0.1.   0.1) | 0.3 (  0.3.    0.3) | 0.0 (  0.0,   0.0) |
|  |  | Denmark | 0.2 (  0.2,   0.2) | 0.1 (  0.0.   0.2) | 0.1 (  0.0.    0.2) | 0.1 (- 0.1,   0.2) |
|  |  | Finland | 0.8 (  0.8,   0.9) | 0.3 (  0.2.   0.4) | - | - |
|  |  | Austria | 0.4 (  0.3,   0.4) | 0.1 (  0.1.   0.2) | 0.2 (  0.1.    0.2) | 0.1 (- 0.1,   0.2) |
|  |  | Spain | 0.3 (  0.3,   0.4) | 0.1 (  0.0.   0.2) | 0.4 (  0.2.    0.6) | -0.1 (- 0.2,   0.1) |
|  |  | Estonia | 0.7 (  0.6,   0.8) | 0.7 (  0.4.   1.0) | 0.7 (  0.4.    1.0) | - |
|  |  | Lithuania | 1.3 (  1.2,   1.4) | 0.7 (  0.2.   1.1) | 0.7 (  0.2.    1.1) | - |
|  | Women | | | | | |
|  |  | Europe (mean) | 0.1 (  0.1,   0.1) | 0.0 (  0.0.   0.0) | 0.1 (  0.1.    0.1) | 0.0 (  0.0,   0.0) |
|  |  | Denmark | 0.1 (  0.1,   0.1) | 0.0 (  0.0.   0.1) | 0.0 (- 0.1.    0.1) | 0.0 (- 0.1,   0.1) |
|  |  | Finland | 0.5 (  0.5,   0.5) | 0.2 (  0.2.   0.3) | - | - |
|  |  | Austria | 0.1 (  0.1,   0.1) | 0.0 (  0.0.   0.0) | 0.0 (  0.0.    0.0) | 0.0 (- 0.1,   0.1) |
|  |  | Spain | 0.1 (  0.1,   0.1) | 0.0 (- 0.1.   0.1) | 0.1 (  0.0.    0.3) | 0.0 (- 0.1,   0.1) |
|  |  | Estonia | 0.1 (  0.0,   0.2) | -0.1 (- 0.3.   0.2) | -0.1 (- 0.3.    0.2) | - |
|  |  | Lithuania | 0.4 (  0.3,   0.4) | 0.2 (  0.0.   0.4) | 0.2 (  0.0.    0.4) | - |

^a^ Differential in partial life expectancy between high and low education groups

^b^ Differential in partial life expectancy between non-manual and manual laborers


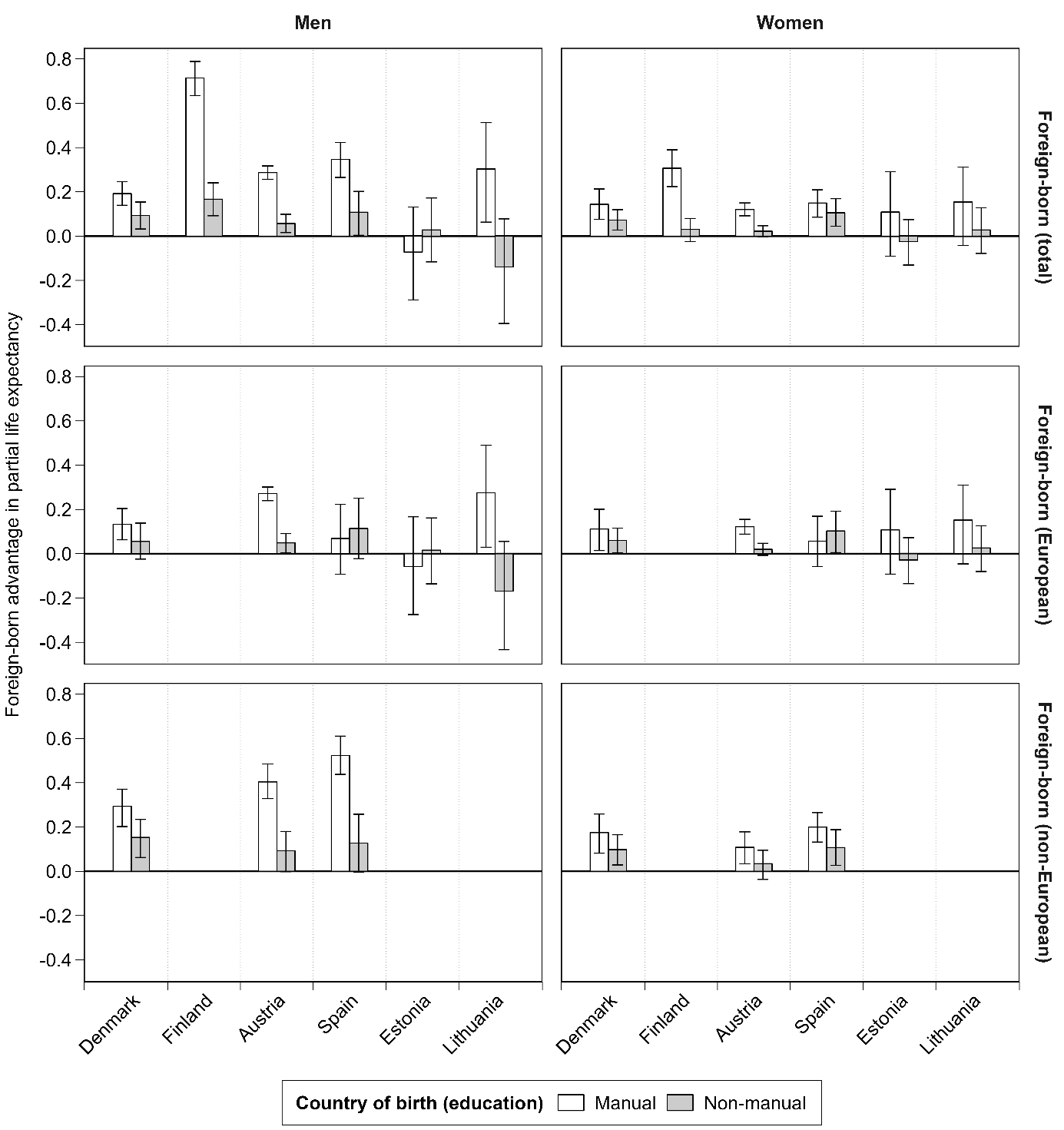


#### Differences in partial life expectancy between foreign-born and native-born populations in each country by occupation, stratified by gender and region of origin. *Note*: Partial life expectancy by occupational group is estimated for ages between 35 and 65.


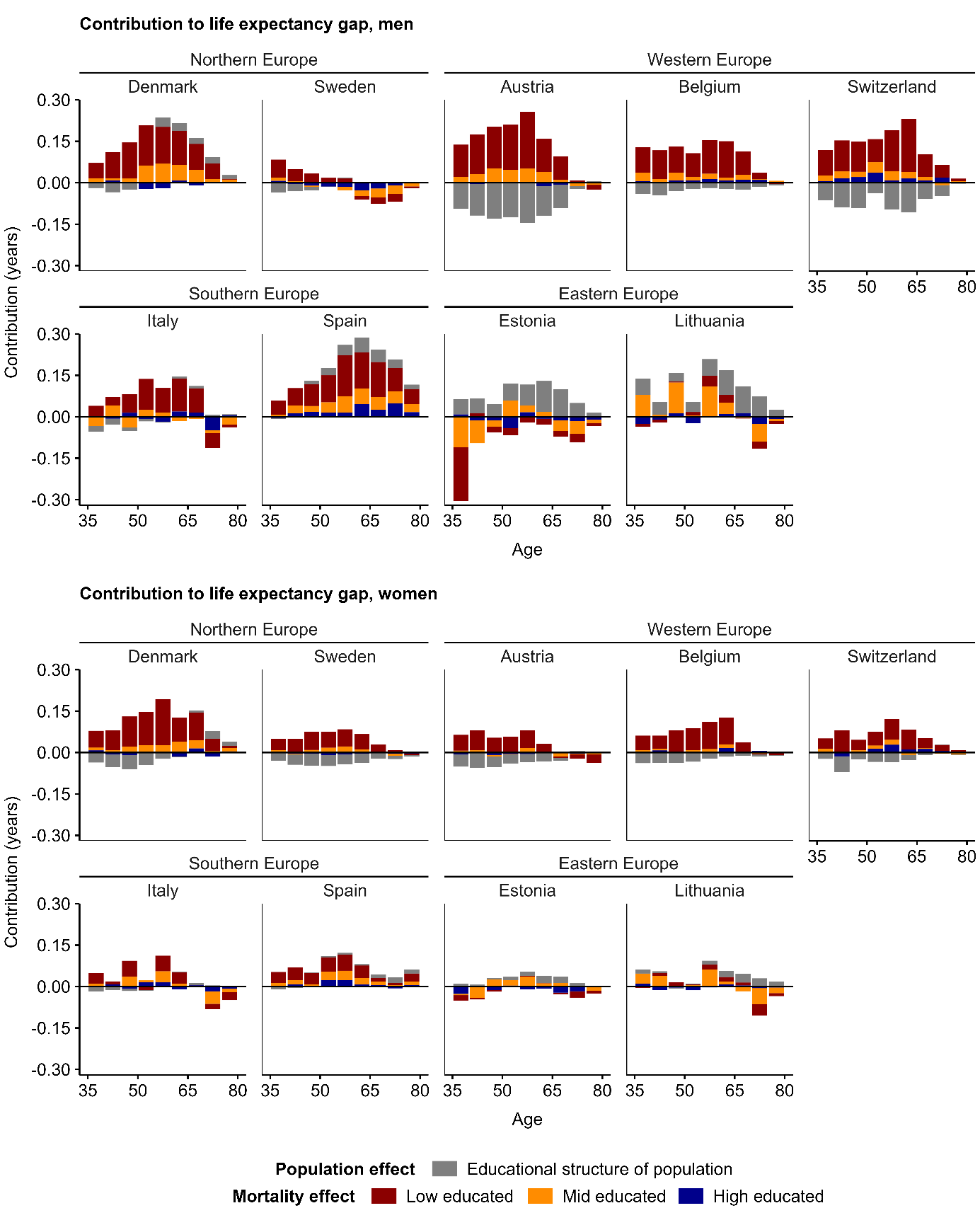


#### Decomposition of the life expectancy gap between foreign-born and native-born populations in each country into the contributions of the mortality and educational structure of the population
